# The Specialist Nurse in European Healthcare 2030: ESNO Congress 2024 Highlights

**DOI:** 10.3390/healthcare12161623

**Published:** 2024-08-15

**Authors:** Alessandro Stievano, Rosario Caruso, Adriano Friganović

**Affiliations:** 1 Department of Clinical and Experimental Medicine, University of Messina, 98100 Messina, Italy; alessandro.stievano@gmail.com; 2 Health Professions Research and Development Unit, IRCCS Policlinico San Donato, 20097 San Donato Milanese, Italy; 3 Department of Biomedical Science for Health, University of Milan, 20133 Milan, Italy; 4 Department of Quality Improvement and Assurance, University Hospital Centre, 10000 Zagreb, Croatia; adriano@hdmsarist.hr; 5 Department of Nursing, University of Applied Health Sciences, 10000 Zagreb, Croatia; 6 Department of Nursing, Faculty of Health Studies, University of Rijeka, 51000 Rijeka, Croatia

**Keywords:** specialist nurses, European healthcare, professional development, ESNO congress 2024, nursing education

## Abstract

The European Specialist Nurses Organization (ESNO), after a series of congresses in Brussels, organised its 6th International Congress in Milan, Italy. The ESNO Congress 2024 focused on “The Specialist Nurse in European Healthcare 2030”, addressing the evolving roles and increasing importance of specialist nurses. The event featured keynote presentations and discussions on enhancing clinical practice through advanced education, bridging policy–practice gaps, and improving working conditions. The ESNO Declaration emphasised lifelong learning, harmonised qualification recognition, and interdisciplinary collaboration. A dynamic hackathon preceded the congress, generating innovative solutions to pressing nursing challenges. New inductees of the ESNO Fellowship Program were celebrated. The congress highlighted critical advancements and set a strategic roadmap for the future of specialist nursing in Europe.

## 1. Introduction

The European Specialist Nurses Organisation (ESNO) is a non-profit organisation dedicated to enhancing communication and cooperation among European Specialist Nurses Organisations and their members [1]. ESNO represents these organisations’ mutual interests and benefits to the broader European community, emphasising public health improvement [1,2]. As a collaborative platform, ESNO provides a “home” for European nurses across various specialities, facilitating professional engagement in European projects, fostering new collegial relationships, and promoting the exchange of knowledge and insights.

ESNO actively promotes and represents the interests of specialist nurses in Europe. ESNO seeks recognition under the Directive of Professional Qualification through collaboration with stakeholder organisations and the utilisation of advanced science [2]. The organisation contributes to effective cooperation between health professionals, organisations, institutes, and agencies, establishing professional standards for education and ongoing development. ESNO implements long-term strategies and policies to advance professional development and health projects across Europe [1,2]. This modus operandi is achieved through consultation with member organisations, partners, and management associates and by participating in numerous European Union institutions, forums, and platforms alongside health workforce partners [3].

The ESNO network encompasses a wide array of nursing specialisations, including clinical work, research, education, leadership, and standardisation in hospitals, communities, and universities.

The ESNO Congress 2024 focused on “The Specialist Nurse in European Healthcare 2030”. It was organised after a series of congresses in Brussels and is the 6th International Congress. The topic of the congress is particularly timely given the evolving roles and the increasing importance of specialist nurses in meeting Europe’s future healthcare needs [4]. Over the past decade, there has been an ongoing debate across Europe regarding the necessity and recognition of specialised nurses [1,4,5]. The congress addressed these crucial discussions, providing a platform for stakeholders to deliberate on the future trajectory of specialist nursing and its impact on the healthcare system.

Sharing the highlights of ESNO Congress 2024 provides valuable insights into the latest developments, challenges, and innovations in specialist nursing. The congress was attended by 350 participants from all the continents worldwide. The congress served as a platform for discussing the competencies, education requirements, and regulatory frameworks essential for specialist nurses. This conference report underscores the necessity for cohesive policies, advanced education, and robust professional development frameworks to support specialist nurses in delivering high-quality, patient-centred care across Europe by sharing the highlights of the congress, which was held in Milan between 5 and 7 June 2024.

## 2. The ESNO Declaration: Enhancing the Role of Specialist Nurses towards 2030

At the ESNO Congress 2024, a pivotal moment was the presentation of the ESNO Declaration, titled “Elaborating on the Role of Specialist Nurses and Advanced Level in European Healthcare Towards 2030” [6]. This declaration outlines a strategic roadmap for advancing specialist nurses in Europe, addressing critical challenges and setting forth comprehensive recommendations to enhance their roles and impact on the healthcare system, and represents the context of the congress [7,8,9,10].

The ESNO Declaration emphasises the need to strengthen professional education and development for specialist nurses. It advocates for enhancing their professional education and promoting continuing professional development to improve staff retention and ensure the delivery of high-quality care [11,12,13,14]. The declaration highlights the importance of fostering lifelong learning to keep pace with medical advancements and maintain clinical competence.

To facilitate seamless mobility and ensure consistent standards across the European Union, the declaration calls for developing a harmonised framework for recognising certificates, diplomas, and degrees. This call includes implementing a European framework for automatically recognising specialist nursing qualifications to streamline the recognition process and maintain high standards.

Improving financial and working conditions for specialist nurses is another critical aspect of the declaration, according to the current evidence [15,16,17,18]. It recommends offering competitive salaries and benefits that reflect specialist nurses’ competencies, knowledge, skills, and contributions. Creating positive and supportive work environments by ensuring reasonable workloads, manageable shift schedules, and safe working conditions is essential to prevent burnout and job dissatisfaction [19,20,21,22,23,24].

The declaration also emphasises the importance of promoting professional growth and mobility [25,26,27]. It calls for the provision of clear career pathways and opportunities for professional growth to attract and retain specialist nurses in the healthcare workforce. Addressing financial considerations by offering financial assistance, relocation support, and competitive compensation packages is crucial to promoting effective professional engagement in European health programs [28,29].

Integrating green and digital awareness into healthcare curricula is highlighted as a necessary step to promote sustainability and digital literacy in the sector [30,31]. The declaration advocates for the harmonisation of prescribing practices and educational standards for nurses across Europe to optimise patient care and recognise the expanding role of nurses in pharmaceutical healthcare delivery.

Finally, fostering interdisciplinary collaboration among healthcare professionals is emphasised [32,33,34,35]. The declaration calls for shared decision-making, clear communication channels, and interdisciplinary education and training opportunities to enhance collaboration and improve patient outcomes [35,36,37].

## 3. ESNO’s Hackathon: 5 June 2024

On 5 June 2024, preceding the ESNO Congress, a dynamic hackathon was held in Milan, Italy, under the theme “Solving the Nurses Health Workforce Crisis in One Day: Are You Ready for the Challenge?” This hackathon has become a cherished tradition, bringing together nurses, healthcare professionals, and stakeholders to collaboratively address pressing issues within the nursing profession and healthcare system. The key themes included balancing clinical practice under pressure, bridging the gap between policy and practice, ensuring quality care amid staffing shortages, and enhancing nurses’ public image and influence.

Participants were organised into moderated tables, each dedicated to one of the nine topics. The discussions were designed to be inclusive, with moderators ensuring that all voices were heard and an observer/reporter documenting the insights. The process emphasised brainstorming, evaluating ideas, and developing feasible solutions that could be implemented locally to avoid generality and loss during the process.

The hackathon addressed several critical topics affecting the nursing workforce. These included balancing clinical practice under reduced conditions by devising strategies to maintain quality care amidst constrained resources and high work demands. Another significant theme was bridging the policy–practice gap with proposals to integrate practical insights into policymaking and overcome policy and clinical practice conflicts. The discussion also covered quality care during staffing shortages, focusing on innovative solutions to uphold evidence-based practices and reduce medical errors under pressure.

Explorations were made into the dichotomy between public and private health sector quality, emphasising career progression, work conditions, and societal connections within the public sector. Empowering nurses in management roles was another key topic, with participants formulating solutions to give nurses more responsibility and authority, thereby addressing the challenges posed by rigid protocols. Additionally, the hackathon addressed the need to reshape public perception of the nursing profession through media and nursing profession profiling, reflecting the authentic and diverse nature of nursing.

Supporting nurses in navigating media platforms and fostering acceptance as trusted professionals and influencers was a topic of discussion under the theme of nurses as influencers. Participants also explored strategies to elevate nurses from end-users to leaders in health technology innovation. Lastly, enhancing nursing resilience in crisis response was discussed, focusing on innovative solutions and interdisciplinary collaboration to improve preparedness and resilience.

The hackathon concluded with presentations of the proposed solutions, which were compiled into a report to be shared with the European institutional representatives. These outcomes aim to influence policy and practice, enhancing the role and impact of specialist nurses in European healthcare. The ESNO Hackathon 2024 exemplified the nursing community’s collective effort and innovative spirit, setting a solid foundation for the discussions and initiatives of the subsequent ESNO Congress.

## 4. ESNO Congress 2024: Highlights from Day 1

The ESNO Congress 2024 commenced with an official opening ceremony featuring greetings from the authorities involved, setting the tone for the event. The development of clinical practice through education at the doctoral level was highlighted, emphasising the importance of advanced education in enhancing clinical competencies. Another presentation discussed the evolving roles, positions, and responsibilities of nurses in specialities and advanced roles, highlighting the increasing importance of these roles in modern healthcare.

Academia’s perspective on nursing specialism was explored, providing insights into how academic research and teaching could support and advance nursing practices. The impact of policy and economy on specialist nursing in the European health environment was also addressed, underscoring the need for supportive policies and economic frameworks to enhance nursing practices. The future of nursing within the new health ecosystem and leadership opportunities was discussed, looking at how nurses could adapt and lead in a rapidly changing healthcare landscape. The keynote presentations concluded with an engaging discussion involving participants and speakers, fostering a rich exchange of ideas and perspectives.

The sessions explored the focus on developing nursing specialisation proficiency through innovative approaches such as evidence-based collaborative online international learning. Discussions also covered case management in palliative care in oncology, the competencies required for nurse managers to lead innovation processes in healthcare organisations, and innovative approaches to nurse–patient communication to support therapeutic relationships. Advanced neuroscience nursing competencies for clinical nurse specialists and the application of the balanced scorecard model to map the core competencies of ward managers were also examined.

Another topic focused on the balance between qualification and overqualification in nursing. Topics included the use of diaries and pedometers for treatment adherence in patients with chronic kidney failure, teaching and learning in theoretical–practical courses for non-European nurses, and skills management for new nurses in the operating room. The clinical and relational skills of nephrology and dialysis nurses were discussed, along with the quality and item analysis of student-generated multiple-choice questions and the validation of perceived overqualification scales in nursing.

Innovations and challenges in European healthcare were discussed in a separate session. Topics included the implementation of Early Warning Scores for veterinary patients, the use of synbiotics and probiotics in diabetes management, and the adherence to hand hygiene practices in healthcare facilities. Discussions also covered healthcare workers’ perceptions of personal protective equipment use from a green perspective and the establishment of a European network for nurses in rehabilitation.

Another track explored nursing’s role in addressing European healthcare challenges. Studies presented included self-care among refugees and asylum seekers, nurses’ awareness in managing chronicity and polypharmacy, and the involvement of nurses in COVID-19 vaccination campaigns. Other topics included the experience of frail elderly patients in emergency departments, the measurement equivalence of the Self-Care of Chronic Illness Inventory over time, and capacity building in nursing informatics competencies.

Sessions also included a focus on the nursing workforce and organisational well-being. Discussions included testing a conceptual framework for nursing organisational well-being, workforce challenges in Slovakia, and the Joint Action HEROES project aimed at meeting health challenges. A roundtable discussion on the future developments of nursing specialism brought together various perspectives, leading to a comprehensive exploration of the topic. The first day concluded with a presentation on the changing paradigms in student nursing and retention criteria in a European context, followed by closing remarks.

## 5. ESNO Congress 2024: Highlights from Day 2

The second day began with a welcome address, followed by presentations on clinical evidence in healthcare and the role of nurse specialists in promoting policy at various levels. The development of advanced nursing practice in different global contexts and the importance of competent nurse professionals in the vaccination domain were explored.

Parallel sessions continued to focus on diverse topics. Presentations included a prediction model for intensive care unit delirium, the association between nursing diagnoses and intra-hospital patient transfers, and the role of resilience in children with autism. The evolving role of respiratory nurses and the care of children and adolescents with gender dysphoria were also discussed.

Another session focused on humanising care and person-centred managerial decisions. Presentations highlighted a model of humanising care, revolutionising healthcare through managerial decisions, and caregiver contributions to patient self-care behaviours. The emergency nurses’ perception of feedback on interventions and case management activities in palliative care was also examined.

Further discussions included nursing leadership styles and error management culture, competencies required by ambulance nurses, and the role of nurses in paediatric haematology and oncology. The impact of nursing leaders on nursing-sensitive outcomes and the relationship between work-related stress, workloads, and surgical site infections were explored. The nursing workload in intensive care units was also assessed.

A workshop on nursing and vaccination addressed the roles, responsibilities, autonomy, and leadership in vaccination. The debate focused on vaccination nurse education and curricula building, particularly concerning influenza.

Afternoon sessions covered technical and evidence advancements within nursing practice. Topics included specialised nurse-led care of chronic wounds, integrating social determinants of health into nursing practice, and the use of ultrasound in brain trauma patients as an early indicator of increased intracranial pressure. The congress concluded with a roundtable discussing the experiences, opportunities, storytelling, challenges, and suggestions for advancing and specialising in nursing. The closing ceremony summarised the key takeaways and highlighted the path forward for specialist nursing in European healthcare.

## 6. ESNO Fellowship Program

During the ESNO Congress 2024, the new inductees of the ESNO Fellowship Program were celebrated at a gala dinner on 6 June. This event honoured their achievements and welcomed them into a global community dedicated to transforming healthcare systems. The ESNO Fellowship Program is designed for nursing leaders in education, management, practice, and research. It aims to empower European specialist nurses through advanced professional development, leadership opportunities, interdisciplinary collaboration, research, and innovation. The program enhances professional recognition and offers mentorship, international exposure, and contributions to healthcare policy.

## 7. Conclusions

The ESNO Congress 2024 was a significant event that brought together nursing professionals and stakeholders to deliberate on the future of specialist nursing in Europe. The conference highlighted the evolving roles of specialist nurses and the critical need for advanced education, robust professional development frameworks, and cohesive policies to support their integration into the healthcare system. In this context, the ESNO Declaration represented the framework of the discussion and presentations and outlined a strategic roadmap for enhancing the role and impact of specialist nurses. It emphasised the importance of lifelong learning, harmonised recognition of European qualifications, and improved financial and working conditions. The declaration also called for integrating green and digital awareness into healthcare curricula and fostering interdisciplinary collaboration. The ESNO Congress 2024 underscored the nursing community’s collective effort and innovative spirit, highlighting the critical role of specialist nurses in shaping the future of European healthcare. The event provided valuable insights and set a solid foundation for ongoing discussions and initiatives to advance the nursing profession and allow nurses to serve their communities by unlocking their full potential in contributing to the healthcare arena and the broader welfare areas.
